# Clinical Researches in Electro-Surgery

**Published:** 1874-01

**Authors:** A. D. Rockwell, Geo. M. Beard

**Affiliations:** N. York; N. York


Clinical Researches in Electro-Surgery.—*By A. D. Rockwell, A. M., M.D., etc., etc. and Geo. M. Beard, A.M., M.D., etc., etc.*— N. York. William Wood & Co., 1873; 12 mo. cloth, pp. 72.

Formerly the medical profession were led to cherish the highest hopes of the thereputical powers of electricity in the control of disease, and especially the nervous disorders. These hopes appeared, at the time, well grounded in inductive physiology. The office of every progressive practitioner was furnished with its Galvanic, Electro-Magnetic or Faradaic battery, which was brought into requisition in every promising case. The reaction is well known; the dilapidated machines which escaped from the wreck of devastating war, lie disabled upon the topmost shelves, thickly covered with the dust of years.

Fresh, within the memory of living men, is the recollection of the brilliant promise of Liebig's analysis of soils; agricultural scientists, everywhere, gazed in wonder upon the supposed philosopher's stone, which was to transmute, by its touch, the ignoble earth into the noblest of glittering metals. It was currently believed, by the more credulous and imaginative, that the alchemists magical art had, indeed, discovered a road to the *elixir vitæ*, the great panacea, which was to cure every agricultural ill, and cause the desert to blossom as the rose; and now, soil analysis, as a diagnostic measure, is numbered with the memories of the past. A few years ago, the President of the Royal Agricultural Society remarked in his annual address: "Chemistry has done one thing for agriculture, and one only—i. e., it has dissolved bones with sulphuric acid."

Though the fond dreams of the great Chemist have not been realized in their original fulness, who can estimate the stupendous results which have followed and will follow, the simple solution of bones by the chemical action of oil of vitriol. The question may be pertinently asked, has medicine received at the hands of the Electricians, a substantial gift, comparable to this single contribution of the Chemists to Agriculture?

We have hope that the future may yet develop something more substantial and enduring. The field of investigation of electrical surgery seems promising, and we welcome this little brochure as a step in that direction.

The little volume is neat and attractive; we regret that we cannot name *the price* for the information of those who may wish to order it.

---

We have received a prospectus for yet another scientific journal, to be edited by George M. Beard, A.M., M.D., of New York, under the title of the *Archives of Electrology and Neurology*. This is an age of specialties and specialists, and one of marvelous progress in science, as well as in art. Notwithstanding all the efforts of the profession to frown down specialism in its members, and however much we may lament the letting down from the dignified snuff-box and gold-headed cane of our fathers, no one who attentively reads the signs of the times can fail to perceive that these things are passing away. Progress is the watchword of today; and whether we will or no, we must soon stand aside or be trampled over. Give the specialists a chance, say we.

---

The January Number of *The Galaxy* comes to our table full of good things for the New Year. Elsewhere we make an extract from its department of Scientific Miscellany, and would like to insert two or three others, which we feel would be interesting to our readers, had we room for them. As it is, we cannot do better than suggest a subscription for the whole, directed to Sheldon & Co., 677 Broadway, N. Y., with four dollars enclosed. When it is shown that *The Galaxy* takes the money of our Southern people, and for it returns abuse, then we shall withdraw this advice.

---

Messrs. Mitchell & Sawyer have commenced the publication of a weekly family newspaper in this city, in the laudable hope of bringing back again the halcyon days of "old-fashioned Jeffersonian Democracy."

For some years Col. Sawyer has been the editor of the *Rome Courier*, probably the leading tri-weekly paper of Georgia, and has done as good and valiant service with his pen, as he did some years ago with his sword upon the field of battle. We wish him every success in his new enterprise.

---

We have received the prospectus of Drs. Jewell and Bannister for the early issue of *The Chicago Journal of Nervous and Mental Diseases.* Following closely upon the suspension of Prof. Hammond's Psycological Journal, the proposal seems a bold one, and yet, when we consider the rapidly advancing march of Western Empire, who shall predict failure. We wish the new enterprise every success.

---

We observe that Dr. E. B. Stevens has retired from the editorial charge of the *Cincinnati Lancet and Observer*, and Dr. J. C. Culbertson takes sole charge of its editorial conduct and publication. We welcome Dr. Culbertson to the fraternity.

